# KL1333, a Novel NAD^+^ Modulator, Improves Energy Metabolism and Mitochondrial Dysfunction in MELAS Fibroblasts

**DOI:** 10.3389/fneur.2018.00552

**Published:** 2018-07-05

**Authors:** Kang-Sik Seo, Jin-Hwan Kim, Ki-Nam Min, Jeong-A Moon, Tae-Chul Roh, Mi-Jung Lee, Kang-Woo Lee, Ji-Eun Min, Young-Mock Lee

**Affiliations:** ^1^R&D Center, Yungjin Pharmaceutical, Suwon, South Korea; ^2^Department of Pediatrics, Yonsei University College of Medicine, Seoul, South Korea

**Keywords:** NAD^+^, NQO1, mitochondrial function, MELAS, SIRT1, AMPK, PGC-1α, KL1333

## Abstract

Mitochondrial encephalomyopathy, lactic acidosis, and stroke-like episodes (MELAS), one of the most common maternally inherited mitochondrial diseases, is caused by mitochondrial DNA mutations that lead to mitochondrial dysfunction. Several treatment options exist, including supplementation with CoQ10, vitamins, and nutrients, but no treatment with proven efficacy is currently available. In this study, we investigated the effects of a novel NAD^+^ modulator, KL1333, in human fibroblasts derived from a human patient with MELAS. KL1333 is an orally available, small organic molecule that reacts with NAD(P)H:quinone oxidoreductase 1 (NQO1) as a substrate, resulting in increases in intracellular NAD^+^ levels via NADH oxidation. To elucidate the mechanism of action of KL1333, we used C2C12 myoblasts, L6 myoblasts, and MELAS fibroblasts. Elevated NAD^+^ levels induced by KL1333 triggered the activation of SIRT1 and AMPK, and subsequently activated PGC-1α in these cells. In MELAS fibroblasts, KL1333 increased ATP levels and decreased lactate and ROS levels, which are often dysregulated in this disease. In addition, mitochondrial functional analyses revealed that KL1333 increased mitochondrial mass, membrane potential, and oxidative capacity. These results indicate that KL1333 improves mitochondrial biogenesis and function, and thus represents a promising therapeutic agent for the treatment of MELAS.

## Introduction

Mitochondria are essential organelles that generate most of the energy required by the human body in the form of adenosine triphosphate (ATP). In addition, mitochondria play roles in generation of reactive oxygen species (ROS) and control of cell signaling, cell death, and biosynthetic metabolism (1). Mitochondria also have their own DNA (mtDNA), which encodes various proteins constituting the electron transport chain, the critical machinery for ATP production (2). Therefore, mutations in any of the mitochondrial genes can result in mitochondrial dysfunction, leading to mitochondrial disease. The effects of mitochondrial diseases may occur in any part of the body, but are observed most frequently in tissues that require large amounts of energy, such as brain, heart, and muscle (3).

Mitochondrial encephalomyopathy, lactic acidosis, and stroke-like episodes (MELAS) is a progressive neurodegenerative syndrome associated with multiple organ failure due to mitochondrial dysfunction (4). In most cases, MELAS is caused by maternally inherited mutations in mtDNA, of which the most common is the A3243G mutation in a gene encoding mitochondrial tRNA^Leu(UUR)^ (5). Patients with MELAS experience stroke-like episodes, dementia, headache, vomiting, seizures, lactic acidosis, deafness, growth retardation, and myopathy (6). Supplements such as CoQ10, vitamin C, creatine, sodium pyruvate, and L-arginine are frequently used to treat MELAS, but more efficient and scientifically supported therapies are needed (7).

Nicotinamide adenine dinucleotide (NAD^+^) and its reduced form (NADH) are important regulators of intracellular redox homeostasis, energy metabolism, and many cellular signaling pathways (8). NAD^+^ can be synthesized *de novo* or via a salvage pathway, and can also be generated by conversion of NADH by enzymes such as NAD(P)H:quinone oxidoreductase 1 (NQO1) (9). NAD^+^ acts as a cofactor for several NAD^+^-consuming enzymes, such as sirtuins (SIRT1-7), poly(ADP-ribose) polymerases (PARPs), and cyclic ADP-ribose synthases. Intracellular NAD^+^ levels are decreased in diseases associated with mitochondrial dysfunction or aging (10). Accordingly, elevation of NAD^+^ levels, for example, via application of NAD^+^ precursors or pharmacological compounds, represents a promising strategy for relieving symptoms associated with low NAD^+^ (10). Here, we hypothesized that KL1333 could be used as an effective treatment of mitochondrial diseases including MELAS via its ability to increase the NAD^+^/NADH ratio, which is lower in MELAS due to mitochondrial respiratory chain deficiency (10).

In this study, we found that KL1333 treatment of C2C12 and L6 myoblasts increased NAD^+^ levels via the action of NQO1, and activated the SIRT1/AMP-activated protein kinase (AMPK)/peroxisome proliferator-activated receptor gamma coactivator 1-alpha (PGC-1α) signaling network, which is involved in mitochondrial biogenesis and function. In fibroblasts derived from human patients with MELAS, KL1333 also induced the activation of SIRT1, AMPK, and PGC-1α. In addition, KL1333 increased energy production and mitochondrial function and decreased oxidative stress in MELAS fibroblasts. These results suggest that KL1333 could be used to effectively treat MELAS by modulating intracellular NAD^+^ levels via NQO1. KL1333 is currently in a phase I clinical trial for mitochondrial diseases (randomized, double-blind, placebo-controlled in healthy male volunteers, NCT03056209).

## Materials and methods

### Reagents

KL1333 was synthesized as a derivative of β-lapachone. Recombinant human NQO1 (rhNQO1) protein, NAD, NADH, bovine serum albumin (BSA), Coenzyme Q10 (CoQ10), idebenone, and cytochrome c were purchased from Sigma-Aldrich. CM-H2DCFDA, MitoTracker Green FM, and tetramethylrhodamine methyl ester (TMRM) were purchased from Invitrogen. FLAG-tagged mouse PGC-1α plasmid was purchased from Origen. Nitrocellulose membrane and the Enhanced Chemiluminescence (ECL) system were purchased from Amersham. Anti-AMPK, anti-acetyl-CoA carboxylase (ACC), anti-phospho-AMPK, anti-phospho-ACC, and anti-phospho-serine antibodies were purchased from Cell Signaling Technology. Anti-FLAG and anti-actin antibodies were purchased from Sigma-Aldrich. Anti-NADH dehydrogenase [ubiquinone] 1 alpha subcomplex subunit A9 (NDUFA9), anti-ubiquinol-cytochrome c reductase core protein 1 (UQCRC1), and anti-ATP synthase subunit alpha (ATP5A) antibodies were purchased from Abcam. Anti-succinate dehydrogenase A (SDHA) and anti-cytochrome c oxidase I (COX I) antibodies were purchased from Invitrogen.

### Cell culture

C2C12 mouse myoblasts, L6 rat myoblasts, and HepG2 human hepatocarcinoma cells were obtained from the American Type Culture Collection (ATCC). Human fibroblasts were isolated from a skin biopsy of a healthy person (wild-type, WT) or MELAS patients harboring a heteroplasmic A3243G mutation. Cells were cultured in Dulbecco's modified Eagle's medium (25 mM glucose) supplemented with 10% fetal bovine serum in an atmosphere containing 5% CO_2_ at 37°C.

### NQO1 oxidation assay

NADH oxidation assays were performed with rhNQO1. NQO1 protein (2.5 mU) was mixed with KL1333, CoQ10, or idebenone at various concentrations (0.1, 0.25, 0.5, 1, 2.5, 5, 10, 25, 50, and 100 μM) in 50 mM Tris-HCl (pH 7.5) buffer containing 0.14% BSA. Reactions were initiated by addition of 200 μM NADH, and the change in absorbance at 340 nm was measured over time for 3 min at 25°C (extinction coefficient for NADH [εNADH] = 6,220 M^−1^ · cm^−1^).

### Cytochrome c reduction assay

The reaction medium consisted of 77 μM cytochrome c, 200 μM NADH, and each compound (KL1333, CoQ10, or idebenone; 0.1–100 μM range) in 50 mM Tris-HCl (pH 7.5) buffer containing 0.14% BSA. Cytochrome c reduction activity was measured at 30°C using NADH as the immediate electron donor and cytochrome c as the terminal electron acceptor. Reactions were initiated by the addition of rhNQO1 (5 mU). Activity was calculated as μmol of cytochrome c reduced/mg/min of protein, based on the initial rate of change in OD at 550 nm and the extinction coefficient for cytochrome c (21.1 mM^−1^ · cm^−1^).

### Measurement of NAD^+^/NADH ratio

Intracellular NAD^+^ and NADH levels were measured using the EnzyChrom NAD^+^/NADH Assay Kit (BioAssay Systems). Briefly, cells were homogenized in either 100 μl of NAD^+^ extraction buffer (for NAD^+^ determination) or 100 μl of NADH extraction buffer (for NADH determination). Samples were heated at 60°C for 5 min, and then mixed with 20 μl of assay buffer and 100 μl of the opposite extraction buffer to neutralize the extracts. Next, samples were briefly vortexed, and centrifuged at 14,000 rpm for 5 min. Supernatants were subjected to NAD^+^/NADH assays based on the lactate dehydrogenase cycling reaction, in which the generated NADH reduces a tetrazolium salt to a purple colored formazan product. NAD^+^ and NADH levels were quantified by measuring the increase in formazan at 570 nm using a microplate reader.

To prepare the cell-free enzyme system, 1 μM KL1333 was mixed with 200 μM NADH and 200 μM NAD in 50 mM Tris-HCl (pH 7.5) buffer containing 0.14% BSA (total volume, 200 μl). Reactions were initiated by addition of rhNQO1 (10 mU), and assay mixtures were incubated for 1 h at 37°C. Twenty microliters of reaction mixture were mixed with either 100 μl of NAD^+^ extraction buffer or 100 μl of NADH extraction buffer. Samples were heated at 60°C for 5 min, and then mixed with 20 μl of assay buffer and 100 μl of the opposite extraction buffer to neutralize the extracts. Next, samples were briefly vortexed, and centrifuged at 14,000 rpm for 5 min. Supernatants were subjected to NAD^+^/NADH assays, and NAD^+^ and NADH levels were measured on a microplate reader by monitoring absorbance at 570 nm.

### Measurement of SIRT1 activity

SIRT1 activity was measured using the SIRT1 Activity Assay Kit (Abcam). Cells were seeded into 6 well plates (2 × 10^5^ cells/well). The day after, the medium was removed and cells were treated with 1 or 2 μM KL1333. After 1 h, cells were harvested and lysed in lysis buffer. Reactions were initiated by adding cell lysates to the reaction mixture containing SIRT1 assay buffer, fluoro-substrate peptides (100 mM), and NAD^+^ (100 mM). Fluorescence intensity was measured for 30 min at 2–3 min intervals on a microplate fluorometer (excitation, 350 nm; emission, 460 nm). SIRT1 activity was calculated within the linear range of reaction velocity, and normalized against the protein concentration in WT control cells.

### Luciferase assay

C2C12 myoblasts were seeded into 6 well plates (2 × 10^5^ cells/well). The day after, cells were co-transfected with pGL3-mouse PGC-1α luciferase reporter and pRL-SV40 encoding *Renilla* luciferase using Turbofect transfection reagent (Thermo Scientific). The transfected cells were allowed to stabilize for 24 h, and then treated with 1 μM KL1333 for 24 h. Cell lysates were subjected to luciferase assay using the Dual-Luciferase Reporter Assay System (Promega). Luciferase activity was measured using a luminometer (Anthos Labtec Instrument). Firefly luciferase activity was normalized against *Renilla* luciferase activity.

### Quantification of ATP levels

To measure intracellular ATP levels, the ATP Determination Kit (Invitrogen) was used. Human fibroblasts were seeded into 6 well plates (1.5 × 10^5^ cells/well). The day after, cells were treated with 1 μM KL1333 or 1 μM idebenone for 24 h, and then lysed in 100 μl of cell lysis buffer. Cell lysates were centrifuged at 13,000 rpm for 10 min. ATP levels in the supernatant were measured using a luminometer.

### Determination of intracellular lactate levels

Intracellular lactate levels were measured using the Lactate Colorimetric Assay Kit (BioVision). Human fibroblasts were treated with 1 μM KL1333 for 24 h, and then homogenized in assay buffer. Cell lysates were reacted with reaction mixture containing assay buffer, substrate mix, and enzyme mix, and then incubated for 30 min at room temperature. Lactate levels in each sample were analyzed by monitoring optical density at 450 nm.

### Measurement of intracellular ROS levels

Human fibroblasts were treated with 1 μM KL1333 for 24 h, and then incubated with 2 μM CM-H2DCFDA for 30 min at 37°C. The cells were then washed twice with phosphate-buffered saline (PBS) and resuspended in 500 μl of PBS. ROS levels in each sample were analyzed using a flow cytometer (excitation, 488 nm; emission, 530 nm).

### Measurement of mitochondrial mass and mitochondrial membrane potential

Human fibroblasts were treated with 1 μM KL1333 for 24 h. After treatment, cells were washed with PBS and trypsinized, centrifuged at 400 g for 5 min, and resuspended in PBS. Cell suspensions were mixed with 200 nM MitoTracker Green FM (excitation, 488 nm; emission, 530 nm) for assessment of mitochondrial mass, or 200 nM TMRM (excitation, 488 nm; emission, 585 nm) for measurement of mitochondrial membrane potential. The cells were then stained at 37°C in a CO_2_ incubator for 30 min, washed with PBS, and analyzed using a flow cytometer.

### Immunoprecipitation and western blotting

Cells were lysed in cell lysis buffer containing 50 mM Tris-HCl (pH 7.4), 150 mM NaCl, 5 mM EDTA, 1% NP-40, and protease inhibitor cocktail (Roche). For immunoprecipitation assay, the appropriate antibodies were added to the lysates followed by the addition of protein A/G agarose (Santa Cruz biotechnology), and then incubated overnight at 4°C. Lysates were subjected to sodium dodecyl sulfate polyacrylamide gel electrophoresis (SDS-PAGE) and blotted onto nitrocellulose membrane. After blocking with 5% skim milk, the membranes were incubated overnight at 4°C with primary antibodies. Membranes were washed, incubated with the appropriate secondary antibodies for 1 h at room temperature, and rewashed. Proteins were detected by using the ECL system.

### Real-time PCR

Total RNA was isolated using TRIzol Reagent (Invitrogen) according to the manufacturer's instructions. Complementary DNA was synthesized using M-MLV reverse transcriptase (ELPIS Biotech) at 42°C for 1 h. The mixture was then boiled for 5 min to inactivate reverse transcriptase and quickly chilled on ice. Synthesized cDNAs were analyzed by real-time PCR using an EXPRESS SYBR GreenER (Invitrogen) kit on an IQ5 Real-Time PCR detection system (BioRad). Each value was normalized to 18S rRNA levels.

### Measurement of oxygen consumption rate

Mitochondrial oxygen consumption rate (OCR) was measured using a Seahorse XF24 extracellular analyzer (Seahorse Bioscience). Cultured human fibroblasts were washed and incubated with assay medium (DMEM without sodium bicarbonate) at 37°C in a non-CO_2_ incubator for 1 h. Three baseline measurements of OCR were taken before sequential injection of mitochondrial inhibitors: oligomycin (2 μg/ml), carbonyl cyanide-p-trifluoromethoxyphenylhydrazone (FCCP) (10 μM), and antimycin A (5 μM). OCR was automatically calculated and recorded by the Seahorse XF24 software. Percentage change relative to the basal rate was calculated as the value of the change divided by the average value of the baseline readings. After the assays, cells in each well were lysed by cell lysis buffer, and the protein levels were measured by the Bradford method. OCRs in each sample were normalized against the protein concentration of WT control cells.

### Statistical analysis

Results are expressed as means ± standard error of the mean (SEM). Statistical significance between two groups was determined by using the unpaired Student's *t*-test. Comparisons among several groups were performed by analysis of variance, and statistical significance was calculated by using Dunnett's multiple comparison test.

The statistical significance of differences between groups was analyzed using Student's *t*-test or analysis of variance (ANOVA). Differences were considered to be significant at *P* < 0.05.

## Results

### KL1333 increases NQO1 activity and intracellular NAD^+^/NADH ratio

We investigated the effects of KL1333 on NQO1 in two experiments. First, to determine how fast NADH is oxidized to NAD^+^, we measured the rate of decrease in the concentration of NADH. Second, to determine how fast reduced KL1333 transports electrons to cytochrome c, we measured the rate of cytochrome c reduction. In both experiments, CoQ10 and idebenone, a CoQ10 derivative that accepts electrons from NQO1, were used for comparison (11). The results of the NADH oxidation assays revealed little or no change in the absorbance of idebenone and CoQ10, whereas KL1333 exhibited a much higher NADH oxidation activity than either of the comparison compounds (Table 1). These experiments revealed that KL1333 was significantly more active and potent than idebenone and CoQ10.

**Table 1:** Steady-state kinetic constants of NQO1 with KL1333, CoQ10, and Idebenone.

	**NADH oxidation**			**Cytochrome c reduction**		
	**KL1333**	**CoQ10**	**Idebenone**	**KL1333**	**CoQ10**	**Idebenone**
Km (μM)	0.27	–^*^	2.13	0.79	–^*^	1.76
Vmax (μmol/mg/min)	63.9	–^*^	38.3	312.4	–^*^	68.1

* *No enzymatic activity above the background level could be detected for CoQ10; thus, the steady-state kinetics could not be calculated*.

**Figure 1:**
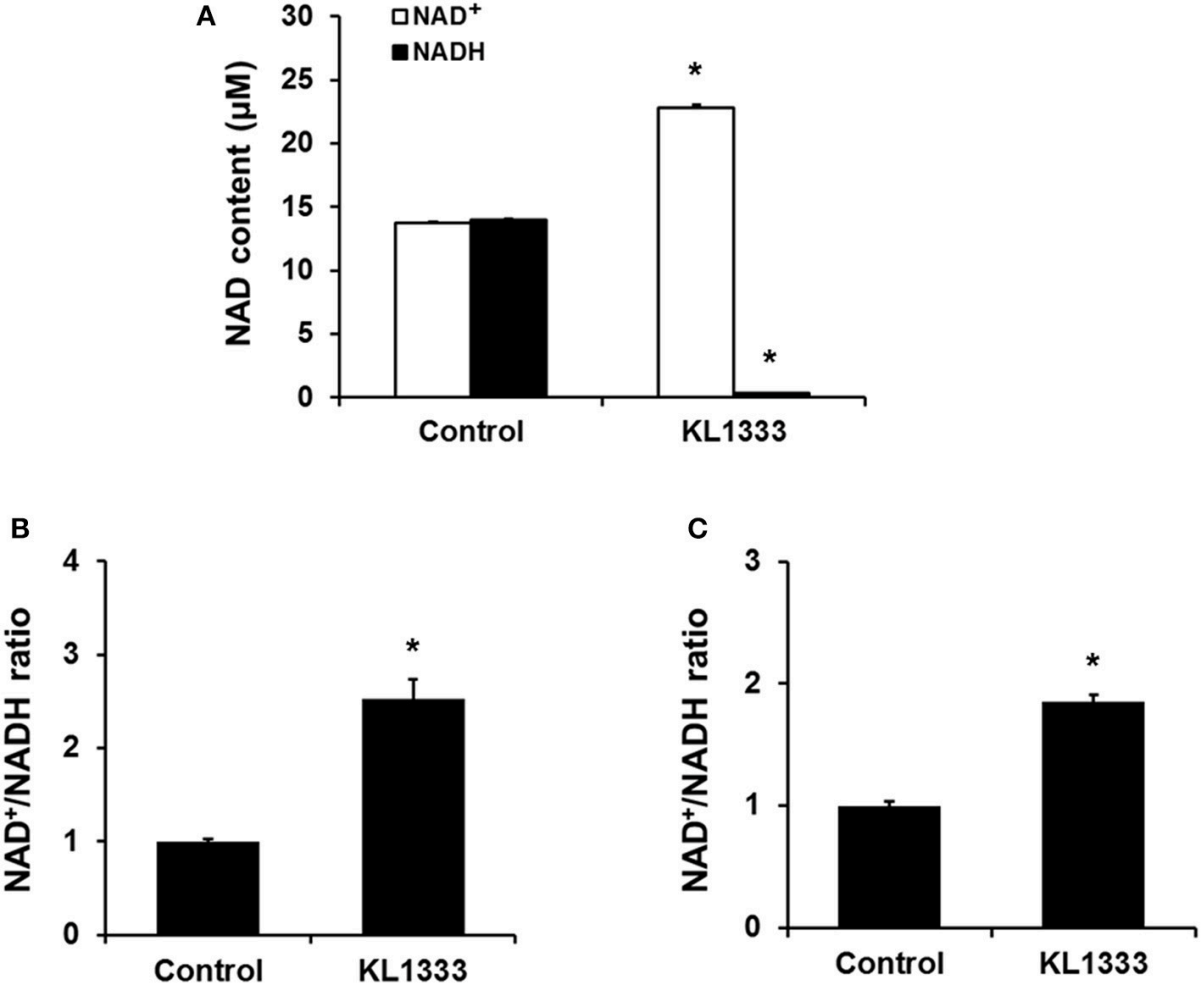
KL1333 increases the intracellular NAD^+^/NADH ratio. **(A)** Reaction mixtures consisting of rhNQO1, NAD^+^, NADH, and KL1333 were incubated at 37°C for 1 h, and then NAD^+^ and NADH levels were measured using a microplate reader. **(B)** C2C12 myoblasts were treated with 1 μM KL1333 for 30 min. Intracellular NAD^+^ and NADH were extracted, and NAD^+^ and NADH levels were measured using a microplate reader. NAD^+^/NADH ratio was calculated based on the concentration of NAD^+^ and NADH. **(C)** L6 myoblasts were treated with 2 μM KL1333 for 30 min. Intracellular NAD^+^ and NADH were extracted, and then NAD^+^ and NADH levels were measured using a microplate reader. NAD^+^/NADH ratio was calculated based on the concentration of NAD^+^ and NADH. Each experiment was repeated four times. Error bars indicate ±SEM. ^*^P < 0.05.

Given that KL1333 increases NQO1 activity, we investigated whether this compound could oxidize NADH to NAD^+^ in cell-free enzyme assays. The results revealed that KL1333 completely converted NADH to NAD^+^ (Figure 1A). Next, we examined the effect of KL1333 on the NAD^+^/NADH ratio in several cell lines. In C2C12 mouse myoblasts, L6 rat myoblasts, and HepG2 human hepatocarcinoma cells, KL1333 significantly increased the intracellular NAD^+^/NADH ratio (Figures 1B,C; Figure S1A). The KL1333-induced increase in the NAD^+^/NADH ratio was blocked by the NQO1-specific inhibitor ES936 (Figure S1B). Taken together, these results indicate that KL1333 promotes oxidation of NADH to NAD^+^ by NQO1.

### Downstream effects on SIRT1, AMPK, and PGC-1α activation

**Figure 2:**
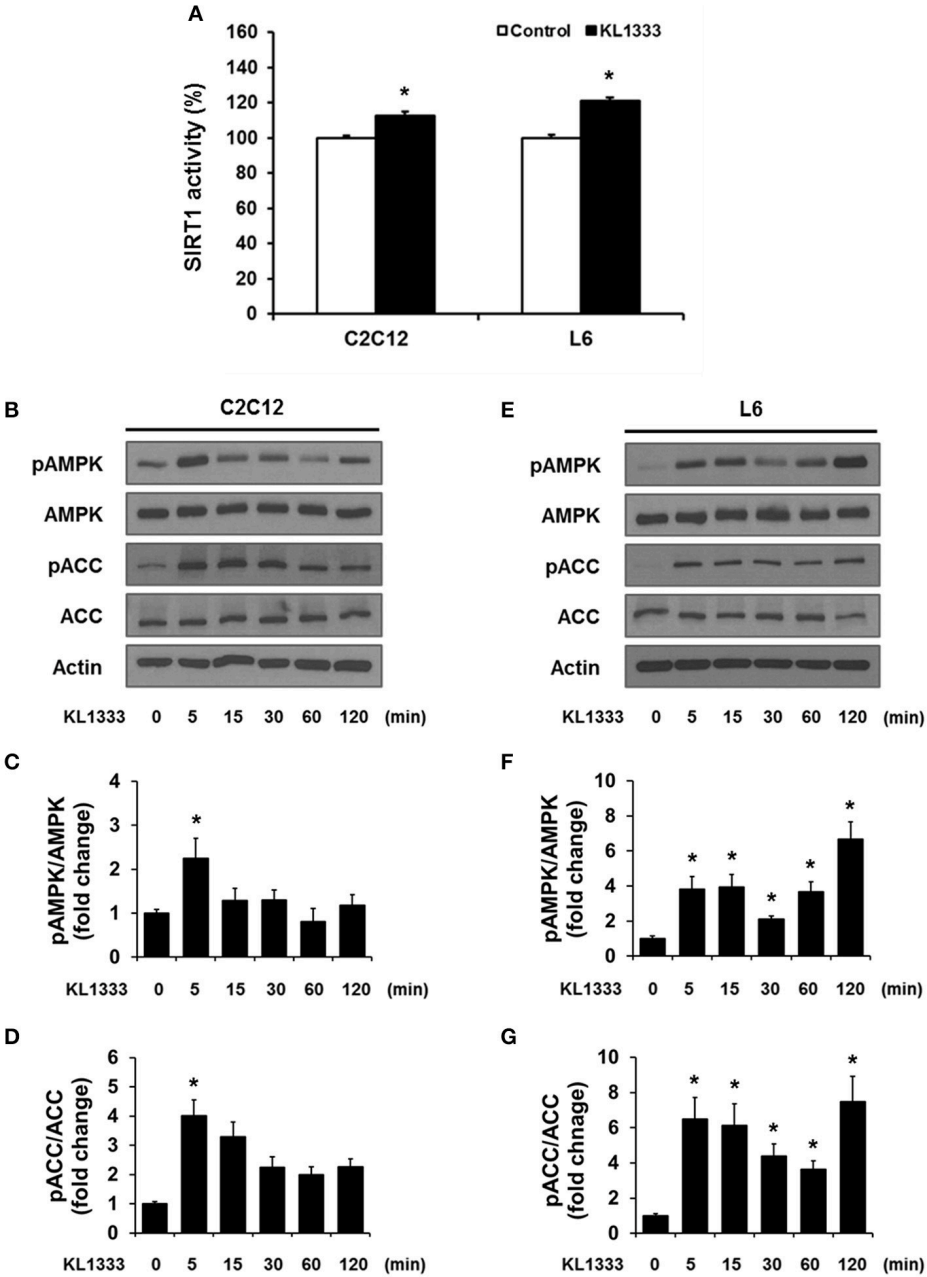
KL1333 activates SIRT1 and AMPK. **(A)** C2C12 and L6 myoblasts were treated with 1 and 2 μM KL1333 for 1 h, respectively. SIRT1 activities in both cell lines were analyzed using a fluorescence-based assay. **(B)** C2C12 myoblasts were treated with 1 μM KL1333 for the indicated times. The activity of AMPK was examined by Western blotting using the indicated antibodies. **(C)** Quantification of pAMPK levels in C2C12 cell lysates using its band density normalized to that of total AMPK. **(D)** Quantification of pACC levels in C2C12 cell lysates using its band density normalized to that of total ACC. **(E)** L6 myoblasts were treated with 2 μM KL1333 for the indicated times. The activity of AMPK was examined by Western blotting using the indicated antibodies. **(F)** Quantification of pAMPK levels in L6 cell lysates using its band density normalized to that of total AMPK. **(G)** Quantification of pACC levels in L6 cell lysates using its band density normalized to that of total ACC. Each experiment was repeated three times. Error bars indicate ± SEM. ^*^P < 0.05.

**Figure 3:**
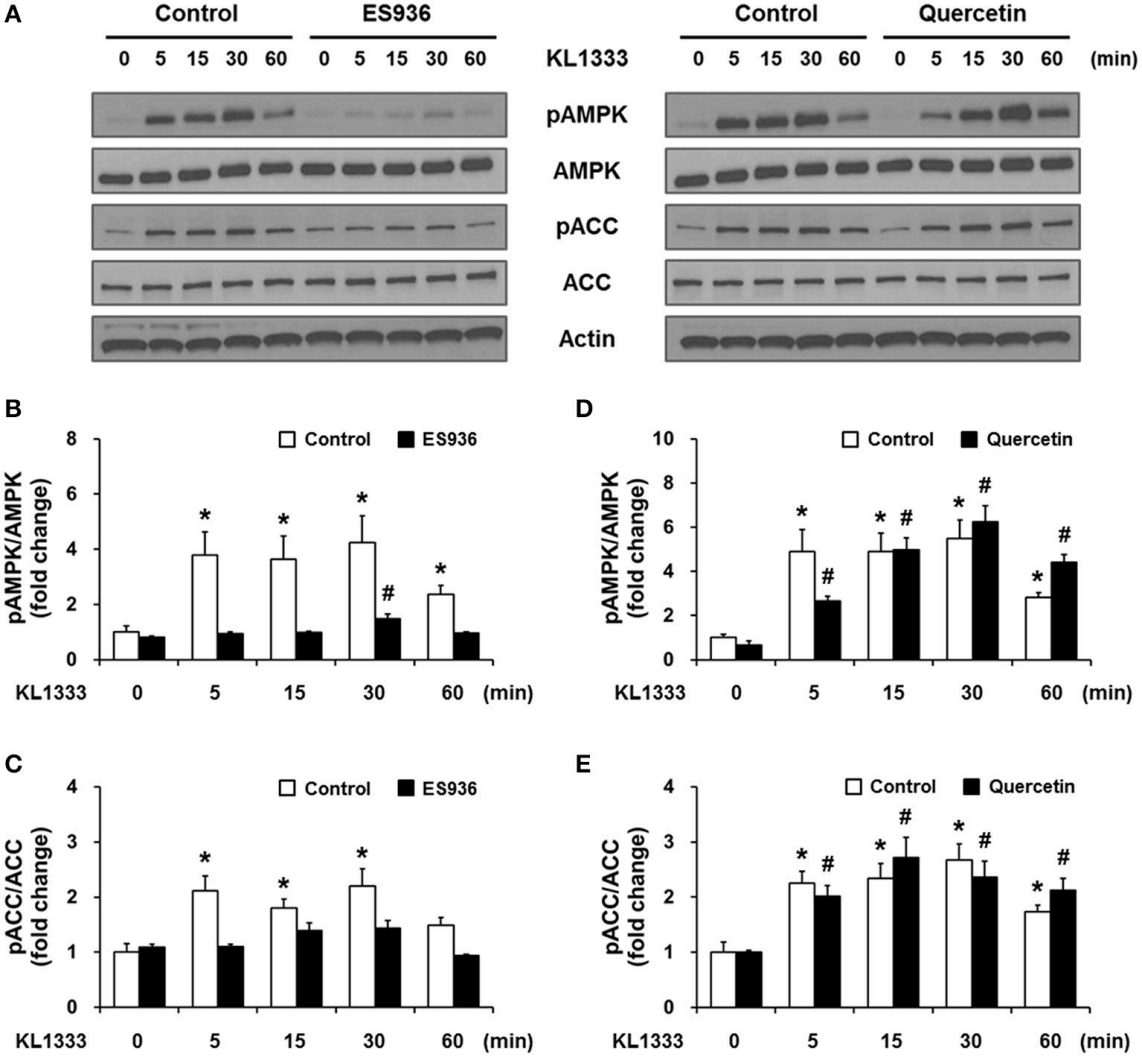
KL1333 activates AMPK in a NQO1-dependent manner. **(A)** L6 myoblasts were pretreated with 250 nM ES936 or 10 μM quercetin for 1 h, and then treated with 2 μM KL1333 for the indicated times. The activity of AMPK was examined by Western blotting using the indicated antibodies. **(B)** Quantification of the pAMPK/AMPK ratio in ES936 and KL1333-treated cells. **(C)** Quantification of the pACC/ACC ratio in ES936 and KL1333-treated cells. **(D)** Quantification of the pAMPK/AMPK ratio in quercetin and KL1333-treated cells. **(E)** Quantification of the pACC/ACC ratio in quercetin and KL1333-treated cells. Each experiment was repeated three times. Error bars indicate ± SEM. ^*^P < 0.05: significance with respect to the control group. ^#^*P* < 0.05: significance with respect to the ES936- or quercetin-treated group.

An increase in the NAD^+^/NADH ratio, such as that induced by KL1333, can activate NAD^+^-dependent deacetylases such as SIRT1. To explore this possibility, we treated C2C12 and L6 myoblasts with KL1333 for 1 h. KL1333 significantly increased SIRT1 activity in both cell lines (Figure 2A). Activation of SIRT1 by elevated intracellular NAD^+^ controls the activity of liver kinase B1, resulting in activation of AMPK, and concurrent dual activation of SIRT1 and AMPK increases intracellular metabolism (12). To determine whether the increase in intracellular NAD^+^ induced by KL1333 leads to the activation of AMPK, we treated C2C12 and L6 myoblasts with KL1333 for the indicated times. KL1333 dramatically induced activating phosphorylation of the catalytic α subunit of AMPK (Figures 2B–G). In addition, AMPK-dependent phosphorylation of ACC was induced by KL1333. Next, we examined the effect of NQO1 and NQO2 inhibitors on KL1333-induced AMPK activation. For this purpose, we pretreated L6 myoblasts with ES936 or quercetin, a NQO2 inhibitor, and then treated with KL1333 for the indicated times. As shown in Figures 3A–E, ES936 dramatically blocked KL1333-induced phosphorylation of AMPK and ACC, whereas quercetin had no effect on the phosphorylation of either protein. These results indicate that KL1333 activates AMPK in a NQO1-dependent manner.

**Figure 4:**
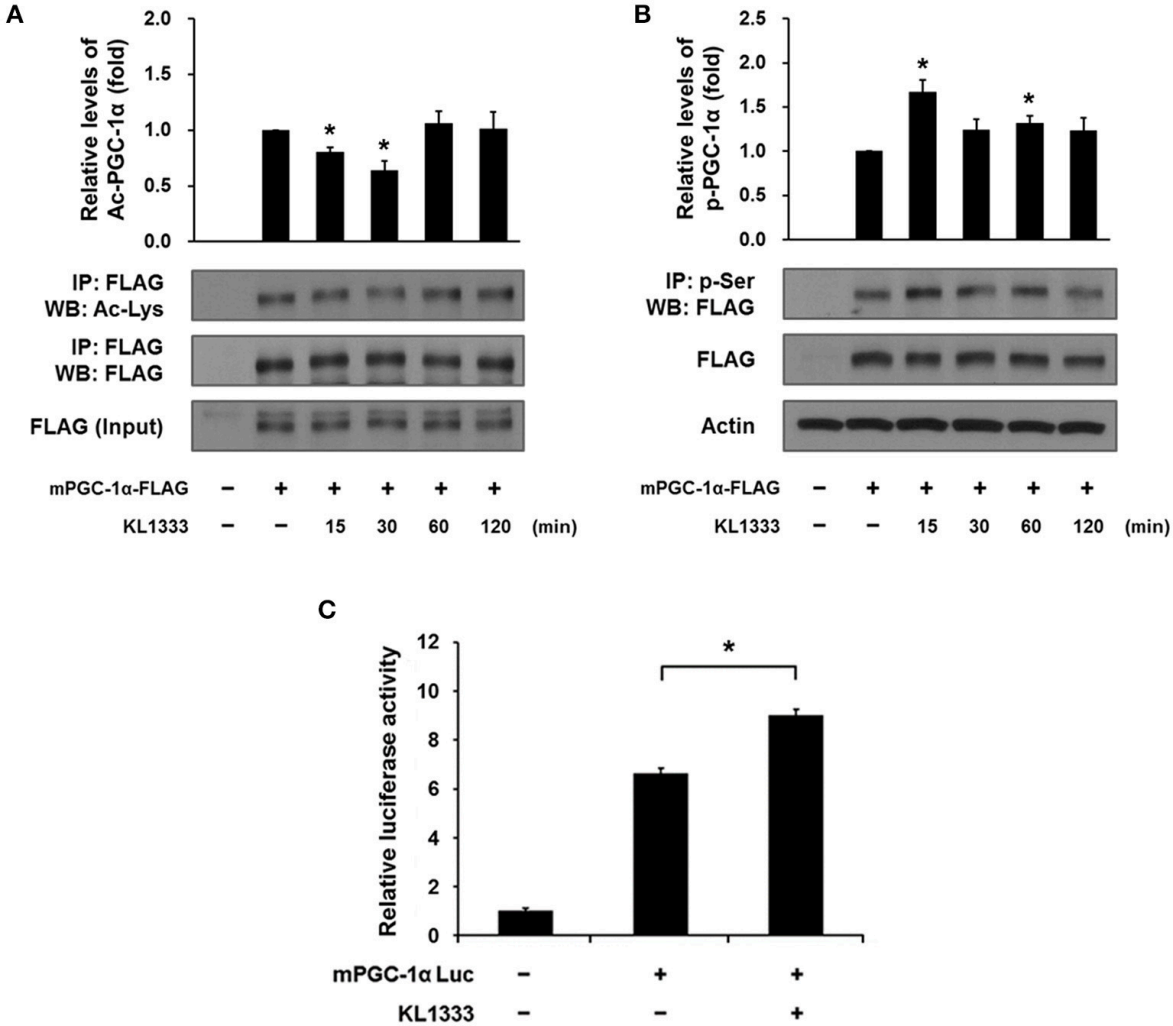
KL1333 induces PGC-1α activation. **(A)** C2C12 myoblasts were transfected with FLAG-tagged mPGC-1α for 48 h, and then treated with 2 μM KL1333 for the indicated times. Cell lysates were subjected to immunoprecipitation using antibody against FLAG. The acetylation levels of PGC-1α were examined by Western blotting using anti-acetyl lysine antibody. Histograms show the levels of acetylation of PGC-1α relative to total FLAG-tagged mPGC-1α under each of the indicated conditions. The experiment was repeated three times. **(B)** C2C12 myoblasts were transfected with FLAG-tagged mPGC-1α for 48 h, and then treated with 2 μM KL1333 for the indicated times. Cell lysates were subjected to immunoprecipitation using antibody against phospho-serine. The phosphorylation levels of PGC-1α were examined by Western blotting using an anti-FLAG antibody. Histograms show the levels of phosphorylation of PGC-1α relative to total FLAG-tagged mPGC-1α under each of the indicated conditions. The experiment was repeated three times. **(C)** Cells expressing PGC-1α luciferase were treated with 1 μM KL1333 for 24 h. Cell lysates were subjected to luciferase assays, and the activity of the PGC-1α promoter was analyzed. The experiment was repeated four times. Error bars indicate ± SEM. ^*^P < 0.05.

Both SIRT1 and AMPK are upstream regulators of PGC-1α, and directly activate PGC-1α by deacetylation and phosphorylation, respectively (12). To determine whether activation of SIRT1 and AMPK induced by KL1333 leads to activation of PGC-1α, we overexpressed a plasmid encoding FLAG-tagged mouse PGC-1α (mPGC-1α-FLAG) in C2C12 myoblasts, treated the PGC-1α-overexpressing cells with KL1333 for the indicated times, and then measured the extent of acetylation and phosphorylation of PGC-1α by immunoprecipitation. The results revealed that KL1333 decreased acetylation of PGC-1α, but increased phosphorylation of the protein (Figures 4A,B). PGC-1α can induce its own expression through an autoregulatory loop (13). Hence, we performed a PGC-1α promoter luciferase assay to determine whether KL1333 increases PGC-1α promoter activity. Indeed, treatment of C2C12 myoblasts with KL1333 for 24 h increased transcription from the PGC-1α reporter by ~35% (Figure 4C).

Taken together, these results suggest that KL1333 increases PGC-1α activity by increasing NAD^+^ levels, which in turn activate SIRT1 and AMPK.

### KL1333 regulates ATP, lactate, and ROS levels in MELAS fibroblasts

**Figure 5:**
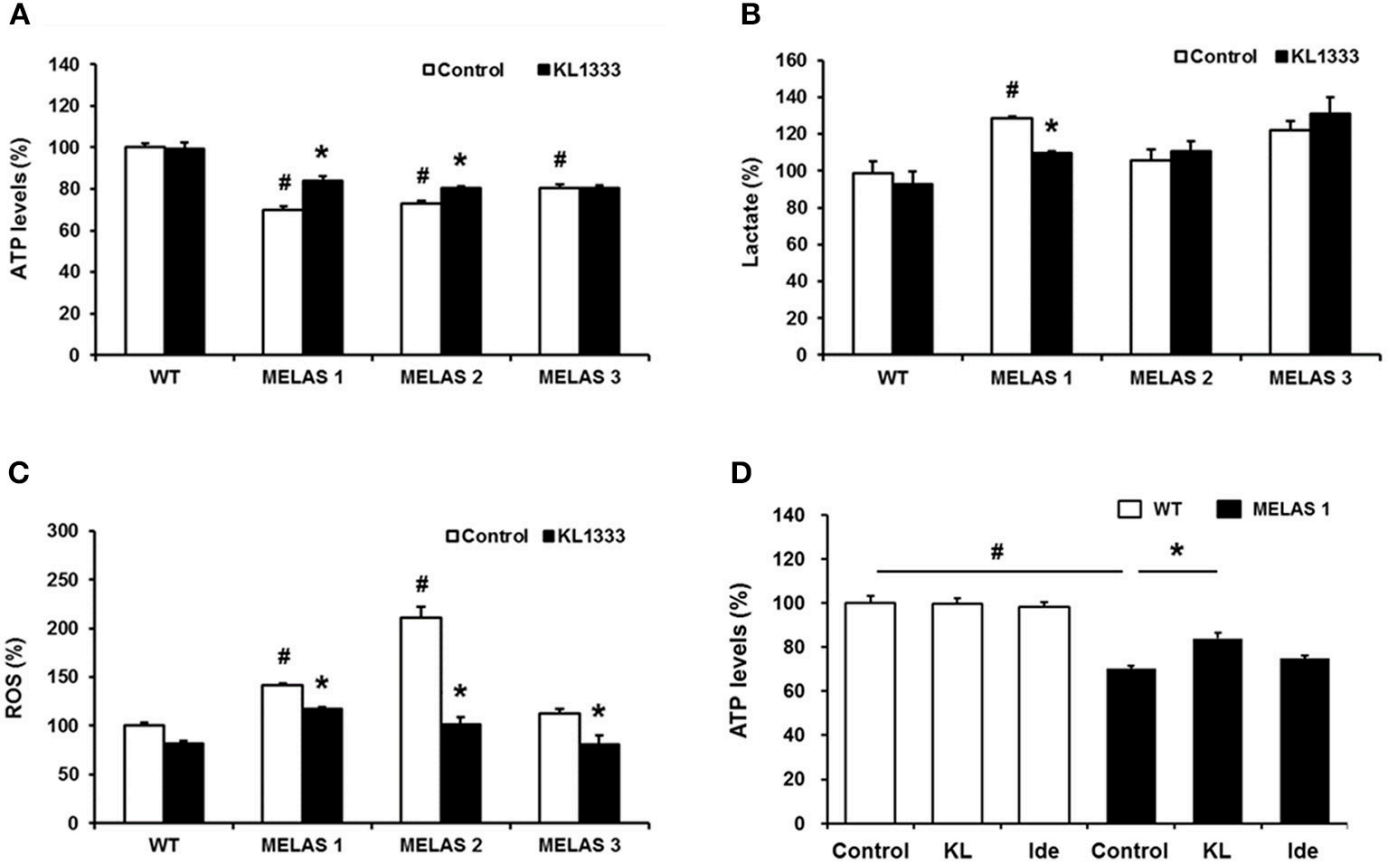
KL1333 regulates intracellular ATP, lactate, and ROS levels in MELAS fibroblasts. Human fibroblasts derived from three patients with MELAS were treated with 1 μM KL1333 for 24 h. **(A)** Cells were lysed in extraction buffer, and intracellular ATP levels were measured. **(B)** Cell lysates were reacted with enzyme mixtures, and intracellular lactate levels were measured. **(C)** Cells were stained with CM-H2DCFDA, and ROS levels were measured using a flow cytometer. **(D)** MELAS 1 fibroblasts were treated with 1 μM KL1333 (KL) or 1 μM idebenone (Ide) for 24 h. Cell lysates were used for measurement of ATP levels. Each experiment was repeated three times. Error bars indicate ± SEM. ^#^*P* < 0.05: MELAS fibroblast vs. WT control. ^*^P < 0.05: presence versus absence of KL1333.

Due to its role in mitochondrial biogenesis and functions related to oxidative phosphorylation (OXPHOS) capacity and oxidative stress (14), PGC-1α is considered to be a therapeutic target for mitochondrial diseases including MELAS. In light of our observation that KL1333 increases PGC-1α activity, we investigated whether KL1333 could restore ATP production in human fibroblasts derived from three MELAS patients harboring the A3243G mutation. The ATP levels in all three fibroblasts were markedly lower than in WT fibroblasts (Figure 5A). However, treatment with KL1333 for 24 h significantly increased the ATP levels in two out of three MELAS patient fibroblasts (MELAS 1 and MELAS 2, but not MELAS 3). Next, we examined the effect of KL1333 on lactate levels in MELAS fibroblasts. As shown in Figure 5B, KL1333 attenuated the elevation in intracellular lactate levels in MELAS 1 fibroblasts, but had no significant effect on lactate in the other two cell lines. Finally, to determine whether KL1333 reduces oxidative stress, we measured ROS levels in MELAS fibroblasts. All three MELAS fibroblast lines contained significantly higher ROS levels than WT fibroblasts (Figure 5C). However, treatment with KL1333 for 24 h markedly decreased ROS levels in all three MELAS fibroblast lines. Taken together, these results indicated that KL1333 can rescue the impairment of energy production and elevated oxidative stress in MELAS fibroblasts in a cell line-specific manner.

Next, because KL1333 exhibited higher activity toward NQO1 than idebenone, we compared the changes in ATP levels treated with each compound. In this experiment, cells were treated with KL1333 or idebenone at the same concentration for 24 h, and then intracellular ATP levels were measured. KL1333 significantly increased ATP levels in MELAS 1 fibroblasts, but not in control fibroblasts, whereas idebenone caused no significant change (Figure 5D).

### KL1333 improves mitochondrial mass and OXPHOS in MELAS fibroblasts

**Figure 6:**
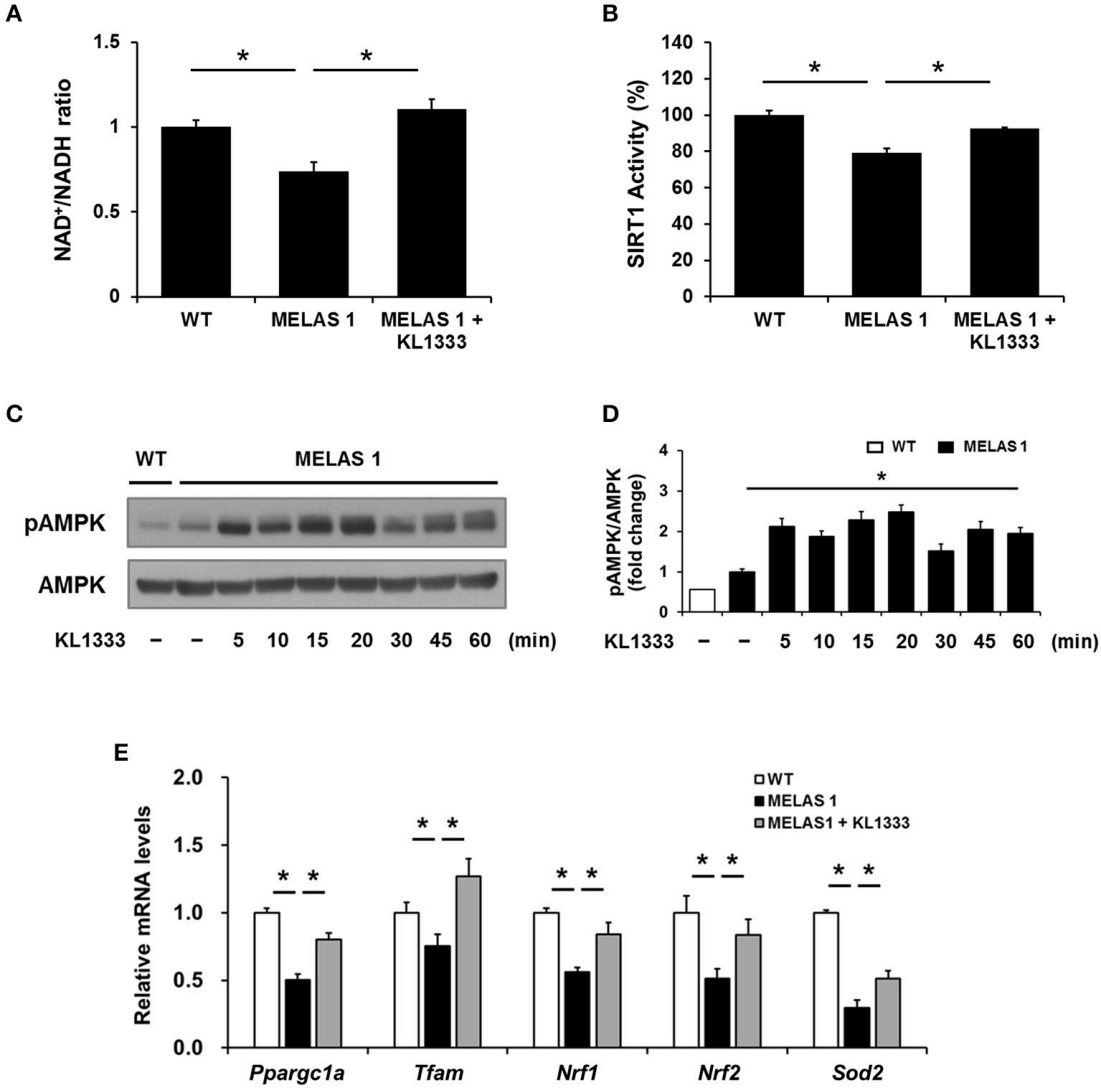
KL1333 increases the NAD^+^/NADH ratio and activates SIRT1, AMPK, and PGC-1α in MELAS fibroblasts. **(A)** MELAS 1 fibroblasts were treated with 1 μM KL1333 for 30 min lysed in extraction buffer, and intracellular ATP levels were measured. Intracellular NAD^+^ and NADH were extracted, and NAD^+^ and NADH levels were measured using a microplate reader. The NAD^+^/NADH ratio was calculated based on the concentration of NAD^+^ and NADH. **(B)** MELAS 1 fibroblasts were treated with 1 μM KL1333 for 30 min. SIRT1 activity was analyzed using a fluorescence-based assay. **(C)** MELAS 1 fibroblasts were treated with 1 μM KL1333 for the indicated times. The activity of AMPK was examined by Western blotting using the indicated antibodies. **(D)** Quantification of pAMPK levels in MELAS 1 fibroblasts using its band density normalized to that of total AMPK. **(E)** MELAS 1 fibroblasts were treated with 1 μM KL1333 for 24 h. The expression of PGC-1α and its target genes were measured by real-time PCR using specific primers for *Ppargc1a, Tfam, Nrf1, Nrf2*, and *Sod2*. Each experiment was repeated three times. Error bars indicate ±SEM. ^*^P < 0.05.

Since KL1333 induces the activation of the SIRT1/AMPK/PGC-1α signaling network through NQO1-mediated oxidation of NADH to NAD^+^ in C2C12 and L6 myoblasts, we investigated whether KL1333 also increases the NAD^+^/NADH ratio and activates SIRT1, AMPK, and PGC-1α in MELAS fibroblasts. As shown in Figure 6A, MELAS 1 fibroblasts had a lower NAD^+^/NADH ratio than WT fibroblasts, but KL1333 significantly increased the NAD^+^/NADH ratio in MELAS fibroblasts above the WT level. In addition, KL1333 induced the activation of SIRT1 and AMPK in MELAS 1 fibroblasts (Figures 6B–D). To examine whether KL1333 activates PGC-1α in MELAS 1 fibroblasts, we investigated the mRNA expression of PGC-1α and its target genes such as *Tfam, Nrf1, Nrf2*, and *Sod2* (14). The mRNA levels of PGC-1α and its target genes were lower in MELAS 1 fibroblasts than in WT fibroblasts (Figure 6E), but KL1333 significantly increased the mRNA levels of these genes.

**Figure 7:**
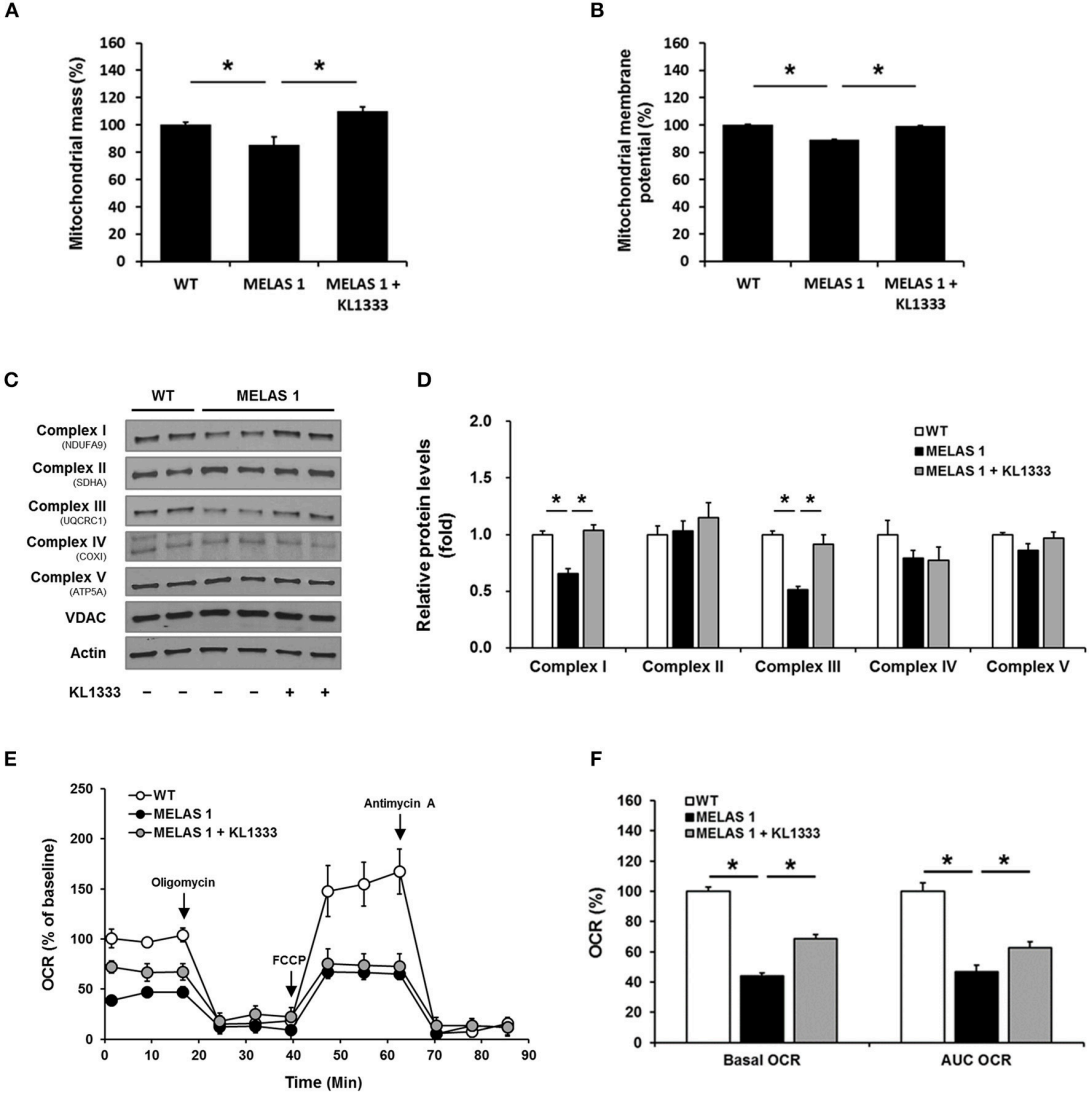
KL1333 improves mitochondrial mass and OXPHOS in MELAS fibroblasts. MELAS 1 fibroblasts were treated with 1 μM KL1333 for 24 h. **(A)** Cells were stained with MitoTracker Green FM, and mitochondrial mass was measured using a flow cytometer. **(B)** Cells were stained with TMRM, and mitochondrial membrane potential was measured using a flow cytometer. **(C)** Protein levels of mitochondrial complex subunits were analyzed by Western blotting using the indicated antibodies. **(D)** Quantification of mitochondrial complex subunit proteins using their band densities normalized to that of VDAC. **(E)** MELAS 1 fibroblasts were treated with 1 μM KL1333 for 24 h, and OCR was measured using the Seahorse XF24 analyzer. Oligomycin, FCCP, and antimycin A were sequentially added at the indicated time points (arrow). **(F)** Basal OCR and AUC OCR are shown in the histogram. Each experiment was repeated three times. Error bars indicate ± SEM. ^*^P < 0.05.

Mitochondrial dysfunction is closely related to impaired energy production and elevated oxidative stress (15). Based on our findings that KL1333 increased ATP generation and decreased ROS levels in MELAS fibroblasts, we examined the effect of KL1333 on mitochondrial mass and OXPHOS. To this end, we treated cells with 1 μM KL1333 for 24 h, and then measured mitochondrial mass. As shown in Figure 7A, MELAS 1 fibroblasts had lower mitochondrial mass than WT fibroblasts, but KL1333 significantly increased mitochondrial mass in MELAS 1 fibroblasts above the WT level. In addition, analysis of mitochondrial membrane potential revealed that KL1333 could also improve mitochondrial function (Figure 7B).

Deficiencies in OXPHOS proteins are closely associated with mitochondrial dysfunction, and PGC-1α plays an important role in mitochondrial biogenesis, mitochondrial protein expression, and mitochondrial respiratory function (16). Hence, we investigated whether KL1333 increases protein expression of mitochondrial complex subunits and mitochondrial OCR. To evaluate the effect of KL1333 on mitochondrial protein expression, we treated MELAS 1 fibroblasts with 1 μM KL1333 for 24 h. KL1333 significantly increased the levels of mitochondrial complex I and III subunits in MELAS 1 fibroblasts (Figures 7C,D), but had no significant effect on the levels of complex II, IV, and V subunits.

Next, we evaluated the effect of KL1333 on mitochondrial respiration by measuring OCR in WT and MELAS 1 fibroblasts. For this purpose, we treated MELAS 1 fibroblasts with 1 μM KL1333 for 24 h, and then measured OCR in the presence of several compounds that control mitochondrial respiration. Oligomycin, a mitochondrial complex V inhibitor, was used to determine the proportion of OCR utilized for ATP production under basal conditions, and the uncoupler FCCP was used to determine maximal mitochondrial respiration (17). In addition, antimycin A was used to inhibit the flow of electrons through complex III, preventing the oxidation of both NADH and succinate by mitochondria. As shown in Figure 7E, OCR was significantly lower in MELAS 1 fibroblasts than in WT fibroblasts. Basal OCR (baseline minus oligomycin), maximal mitochondrial respiration, and area under the curve of OCR (AUC OCR) in MELAS fibroblasts were about half of those in WT fibroblasts (Figure 7F). However, KL1333 significantly increased basal and AUC OCR compared with untreated MELAS 1 fibroblasts, demonstrating that KL1333 improved mitochondrial respiration.

## Discussion

Cellular energy deficiency resulting from mitochondrial dysfunction is a hallmark of mitochondrial diseases. Defects in mitochondrial OXPHOS, and the increase in glycolysis to compensate for the resultant energy deficiency, cause lactate accumulation and acidosis (18). In addition, elevated levels of ROS from electron leakage in the mitochondrial respiratory chain can further cause mtDNA damage, leading to a vicious cycle of ROS accumulation (19). Therefore, strategies aimed at improving mitochondrial function could provide more fundamental and efficacious therapy for patients with mitochondrial diseases than supplementation with nutrients or antioxidants.

Pharmacological stimulation of several proteins improves mitochondrial biogenesis and function; for example, polyphenolic compounds, such as resveratrol, improve mitochondrial function through the activation of SIRT1, which results in PGC-1α activation (20). AMPK-activating compounds also promote mitochondrial function by increasing the expression of PGC-1α target genes involved in mitochondrial biogenesis (21). In addition, a transcription factor, NF-E2 p45-related factor 2 (Nrf2), is an attractive target for improving mitochondrial function since it regulates antioxidant responses, mitochondrial membrane potential, fatty acid oxidation, and OXPHOS activity (22). Furthermore, compounds targeting mitochondrial uncoupling protein improve metabolic conditions such as diabetes and NASH and neurodegenerative diseases such as Alzheimer's disease in experimental models (23, 24). As increased flux in the electron transport chain induced by mitochondrial uncoupling increases utilization of NADH, modulation of NAD^+^/NADH ratio by other means, such as KL1333 reduction by NQO1, may also have a wider therapeutic potential where the NAD^+^/NADH ratio is perturbed (25, 26).

Recently, the approach of providing precursors of NAD^+^ biosynthesis has attracted a great deal of attention in research on mitochondrial medicine. Elevation of NAD^+^ levels by precursors can improve mitochondrial function. For example, nicotinamide riboside increases mitochondrial biogenesis and whole-body metabolism in mouse models of mitochondrial myopathy and diet-induced obesity (27, 28). Similarly, nicotinamide mononucleotide improves age-related phenotypes in mice, including changes in energy metabolism, physical activity, and insulin sensitivity (29). In addition, NAD^+^-boosting compounds such as inhibitors of PARP and CD38 have been studied as therapeutics for treatment of mitochondrial diseases and metabolic syndrome (30, 31). Here, we demonstrated that increasing NAD^+^ through NQO1 represents a promising therapeutic approach for mitochondrial diseases.

NQO1 has been studied as a molecular target for several diseases related to NAD^+^ decline. The reaction of NQO1 with β-lapachone exerts beneficial effects on symptoms of aging, obesity, hypertension, arterial restenosis, acute pancreatitis, cisplatin-mediated acute kidney injury, and hearing impairment (32–34). In addition, we showed previously that β-lapachone increases energy production and improves mitochondrial function in MELAS cytoplasmic hybrid (cybrid) cells, also via the reaction with NQO1 (35). On the other hand, idebenone, a compound recently approved for treatment of Leber's hereditary optic neuropathy (LHON) disease, increases intracellular ATP production and decreases lactate levels in MELAS cybrid cells in a NQO1-dependent manner (11). In addition, idebenone prevents oxidative stress in human fibroblasts derived from patients with LHON (36). Another NQO1 substrate compound, EPI-743, has been developed as a drug for mitochondrial diseases (37, 38). In this study, we showed that KL1333, an orally available small molecule derived from β-lapachone, increased energy production and improved mitochondrial dysfunction and oxidative stress in MELAS fibroblasts. Compared with idebenone, KL1333 was a more potent substrate for NQO1 and had a stronger effect on ATP levels in MELAS fibroblasts. These results suggest that KL1333 may be more effective than idebenone for treatment of mitochondrial diseases.

Our results show that KL1333 augments mitochondrial biogenesis and functions by upregulating the major mitochondrial regulator PGC-1α, which is a well-investigated target in mitochondrial medicine research due to its roles in mitochondrial function and metabolism; specifically, it activates several transcription factors involved in mitochondrial and metabolic gene expression (14). The activity of PGC-1α can be regulated by post-translational modifications. As shown in Figure 4, we found that KL1333 induces dual activation of PGC-1α through deacetylation and phosphorylation, probably mediated by SIRT1 and AMPK, respectively. SIRT1 and AMPK are major metabolic sensors that play various roles in glucose and lipid metabolism, mitochondrial biogenesis, and transcriptional regulation (12). Therefore, the indication for KL1333, as an activator of the SIRT1/AMPK/PGC-1α signaling network, could be extended to aging-related and metabolic diseases such as neurodegeneration, diabetes, and non-alcoholic fatty liver diseases.

Taken together, our results suggest that pharmacological modulation of NAD^+^ via the action of KL1333 on NQO1 improves energy balance, decreases oxidative stress, and restores mitochondrial functions and could be used to relieve the deleterious effects of mitochondrial diseases.

## Ethics statement

The study was approved by the Institutional Review Board of Gangnam Severance Hospital, Yonsei University College of Medicine. Written informed consent was obtained from all volunteers and patients.

## Author contributions

K-SS, J-HK, J-AM, and Y-ML conceived and designed the experiments. K-SS, J-HK, K-NM, J-AM, T-CR, M-JL, K-WL, and J-EM performed the experiments. K-SS and Y-ML wrote the paper. All authors read and approved the manuscript.

### Conflict of interest statement

K-SS, J-HK, K-NM, J-AM, T-CR, M-JL, K-WL, and J-EM are employees of Yungjin Pharmaceutical Co. Ltd. The remaining author declares that the research was conducted in the absence of any commercial or financial relationships that could be construed as a potential conflict of interest. The reviewer GD and handling Editor declared their shared affiliation.

## References

[1] Vyas S Zaganjor E Haigis MC. Mitochondria and cancer. Cell (2016) 166:555–66. 10.1016/j.cell.2016.07.002 27471965 PMC5036969

[2] Federico A Cardaioli E Da Pozzo P Formichi P Gallus GN Radi E. Mitochondria, oxidative stress and neurodegeneration. J Neurol Sci. (2012) 322:254–62. 10.1016/j.jns.2012.05.030 22669122

[3] Suomalainen A Battersby BJ. Mitochondrial diseases: the contribution of organelle stress responses to pathology. Nat Rev Mol Cell Biol. (2018) 19:77–92. 10.1038/nrm.2017.66 28792006

[4] Lin J Zhao CB Lu JH Wang HJ Zhu WH Xi JY. Novel mutations m.3959G>A and m.3995A>G in mitochondrial gene MT-ND1 associated with MELAS. Mitochondrial DNA (2014) 25:56–62. 10.3109/19401736.2013.779259 23834081

[5] El-Hattab AW Emrick LT Hsu JW Chanprasert S Jahoor F Scaglia F. Glucose metabolism derangements in adults with the MELAS m.3243A>G mutation. Mitochondrion (2014) 18:63–9. 10.1016/j.mito.2014.07.008 25086207 PMC4252755

[6] Sandhu JK Sodja C McRae K Li Y Rippstein P Wei YH Effect of nitric oxide donors on cybrids harbouring the mitochondrial myopathy, encephalopathy, lactic acidosis and stroke-like episodes (MELAS) A3243G mitochondrial DNA mutation. Biochem J. (2005) 391:191–202. 10.1042/BJ20050272 15969653 PMC1276916

[7] Mizuguchi Y Hatakeyama H Sueoka K Tanaka M Goto YI. Low dose resveratrol ameliorates mitochondrial respiratory dysfunction and enhances cellular reprogramming. Mitochondrion (2017) 34:43–8. 10.1016/j.mito.2016.12.006 28093354

[8] Canto C Menzies KJ Auwerx J. NAD^+^ metabolism and the control of energy homeostasis: a balancing act between mitochondria and the nucleus. Cell Metab. (2015) 22:31–53. 10.1016/j.cmet.2015.05.023 26118927 PMC4487780

[9] Oh GS Kim HJ Choi JH Shen A Choe SK Kama A. Pharmacological activation of NQO1 increases NAD^+^ levels and attenuates cisplatin-mediated acute kidney injury in mice. Kidney Int. (2014) 85:547–60. 10.1038/ki.2013.330 24025646 PMC3944666

[10] Srivastava S. Emerging therapeutic roles for NAD^+^ metabolism in mitochondrial and age-related disorders. Clin Trans Med. (2016) 5:25. 10.1186/s40169-016-0104-7 27465020 PMC4963347

[11] Haefeli RH Erb M Gemperli AC Robay D Courdier Fruh I Anklin C. NQO1-dependent redox cycling of idebenone: effects on cellular redox potential and energy levels. PLoS ONE (2011) 6:e17963. 10.1371/journal.pone.0017963 21483849 PMC3069029

[12] Price NL Gomes AP Ling AJ Duarte FV Martin-Montalvo A North BJ. SIRT1 is required for AMPK activation and the beneficial effects of resveratrol on mitochondrial function. Cell Metab. (2012) 15:675–90. 10.1016/j.cmet.2012.04.003 22560220 PMC3545644

[13] Handschin C Rhee J Lin J Tarr PT Spiegelman BM. An autoregulatory loop controls peroxisome proliferator-activated receptor γ coactivator 1α expression in muscle. Proc Natl Acad Sci USA. (2003) 100:7111–6. 10.1073/pnas.1232352100 12764228 PMC165838

[14] Wenz T. PGC-1α activation as a therapeutic approach in mitochondrial disease. IUBMB Life (2009) 61:1051–62. 10.1002/iub.261 19859975

[15] Subramaniam SR Chesselet MF Mitochondrial dysfunction and oxidative stress in Parkinson's disease. Prog Neurobiol. (2013) 106–7:17–32. 10.1016/j.pneurobio.2013.04.004 PMC3742021 23643800

[16] Lehman JJ Barger PM Kovacs A Saffitz JE Medeiros DM Kelly DP. Peroxisome proliferator-activated receptor γ coactivator-1 promotes cardiac mitochondrial biogenesis. J Clin Invest. (2000) 106:847–56. 10.1172/JCI10268 11018072 PMC517815

[17] Ehinger JK Piel S Ford R Karlsson M Sjovall F Frostner EA. Cell-permeable succinate prodrugs bypass mitochondrial complex I deficiency. Nat Commun. (2016) 7:12317. 10.1038/ncomms12317 27502960 PMC4980488

[18] Schon EA DiMauro S Hirano M. Human mitochondrial DNA: roles of inherited and somatic mutations. Nat Rev Genet. (2012) 13:878–90. 10.1038/nrg3275 23154810 PMC3959762

[19] Lagouge M Larsson NG. The role of mitochondrial DNA mutations and free radicals in disease and ageing. J Intern Med. (2013) 273:529–43. 10.1111/joim.12055 23432181 PMC3675642

[20] Sandoval-Acuna C Ferreira J Speisky H. Polyphenols and mitochondria: an update on their increasingly emerging ROS-scavenging independent actions. Arch Biochem Biophys. (2014) 559:75–90. 10.1016/j.abb.2014.05.017 24875147

[21] Herzig S Shaw RJ. AMPK: guardian of metabolism and mitochondrial homeostasis. Nat Rev Mol Cell Biol. (2018) 19:121–35. 10.1038/nrm.2017.95 28974774 PMC5780224

[22] Dinkova-Kostova AT Abramov AY. The emerging role of Nrf2 in mitochondrial function. Free Radic Biol Med. (2015) 88:179–88. 10.1016/j.freeradbiomed.2015.04.036 25975984 PMC4726722

[23] Perry RJ Kim T Zhang XM Lee HY Pesta D Popov VB. Reversal of hypertriglyceridemia, fatty liver disease, and insulin resistance by a liver-targeted mitochondrial uncoupler. Cell Metab. (2013) 18:740–8. 10.1016/j.cmet.2013.10.004 24206666 PMC4104686

[24] Geisler JG Marosi K Halpern J Mattson MP. DNP, mitochondrial uncoupling, and neuroprotection: a little dab'll do ya. Alzheimers Dement. (2017) 13:582–91. 10.1016/j.jalz.2016.08.001 27599210 PMC5337177

[25] Liu D Pitta M Mattson MP. Preventing NAD^+^ depletion protects neurons against excitotoxicity: bioenergetic effects of mild mitochondrial uncoupling and caloric restriction. Ann NY Acad Sci. (2008) 1147:275–82. 10.1196/annals.1427.028 19076449 PMC2645538

[26] Baffy G Derdak Z Robson SC. Mitochondrial recoupling: a novel therapeutic strategy for cancer? Br J Cancer (2011) 105:469–74. 10.1038/bjc.2011.245 21712825 PMC3170958

[27] Khan NA Auranen M Paetau I Pirinen E Euro L Forsstrom S. Effective treatment of mitochondrial myopathy by nicotinamide riboside, a vitamin B3. EMBO Mol Med. (2014) 6:721–31. 10.1002/emmm.201403943 24711540 PMC4203351

[28] Canto C Houtkooper RH Pirinen E Youn DY Oosterveer MH Cen Y. The NAD^+^ precursor nicotinamide riboside enhances oxidative metabolism and protects against high-fat diet-induced obesity. Cell Metab. (2012) 15:838–47. 10.1016/j.cmet.2012.04.022 22682224 PMC3616313

[29] Mills KF Yoshida S Stein LR Grozio A Kubota S Sasaki Y. Long-term administration of nicotinamide mononucleotide mitigates age-associated physiological decline in mice. Cell Metab. (2016) 24:795–806. 10.1016/j.cmet.2016.09.013 28068222 PMC5668137

[30] Felici R Cavone L Lapucci A Guasti D Bani D Chiarugi A. PARP inhibition delays progression of mitochondrial encephalopathy in mice. Neurotherapeutics (2014) 11:651–64. 10.1007/s13311-014-0285-y 24935635 PMC4121448

[31] Escande C Nin V Price NL Capellini V Gomes AP Barbosa MT. Flavonoid apigenin is an inhibitor of the NAD^+^ase CD38: implications for cellular NAD^+^ metabolism, protein acetylation, and treatment of metabolic syndrome. Diabetes (2013) 62:1084–93. 10.2337/db12-1139 23172919 PMC3609577

[32] Hwang JH Kim DW Jo EJ Kim YK Jo YS Park JH Pharmacological stimulation of NADH oxidation ameliorate obesity and related phenotypes in mice. Diabetes (2009) 58:965–74. 10.2337/db08-1183 19136651 PMC2661596

[33] Kim HJ Oh GS Shen A Lee SB Choe SK Kwon KB. Augmentation of NAD^+^ by NQO1 attenuates cisplatin-mediated hearing impairment. Cell Death Dis. (2014) 5:e1292. 10.1038/cddis.2014.255 24922076 PMC4611728

[34] Shen A Kim HJ Oh GS Lee SB Lee SH Pandit A NAD^+^ augmentation ameliorates acute pancreatitis through regulation of inflammasome signaling. Sci Rep. (2017) 7:3006 10.1038/s41598-017-03418-0 28592850 PMC5462749

[35] Jeong MH Kim JH Seo KS Kwak TH Park WJ. β-lapachone attenuates mitochondrial dysfunction in MELAS cybrid cells. Biochem Biophys Res Commun. (2014) 454:417–22. 10.1016/j.bbrc.2014.10.093 25451262

[36] Yu-Wai-Man P Soiferman D Moore DG Burte F Saada A. Evaluating the therapeutic potential of idebenone and related quinone analogues in Leber hereditary optic neuropathy. Mitochondrion (2017) 36:36–42. 10.1016/j.mito.2017.01.004 28093355 PMC5644719

[37] Martinelli D Catteruccia M Piemonte F Pastore A Tozzi G Dionisi-Vici C. EPI-743 reverses the progression of the pediatric mitochondrial disease – Genetically defined Leigh syndrome. Mol Genet Metab. (2012) 107:383–8. 10.1016/j.ymgme.2012.09.007 23010433

[38] Gueven N Faldu D. Therapeutic strategies for Leber's hereditary optic neuropathy: a current update. Intractable Rare Dis Res. (2013) 2:130–5. 10.5582/irdr.2013.v2.4.130 25343117 PMC4204556

