# Supplementary material for: KL1333, a Novel NAD+ Modulator, Improves Energy Metabolism and Mitochondrial Dysfunction in MELAS Fibroblasts
Source: Front Neurol. 2018 Jul 5;9:552. doi: 10.3389/fneur.2018.00552 (PMC6041391; doi:10.3389/fneur.2018.00552)
Supplement: Supplementary file 1 [file Presentation_1.PDF]

Supplementary Data

Figure 1

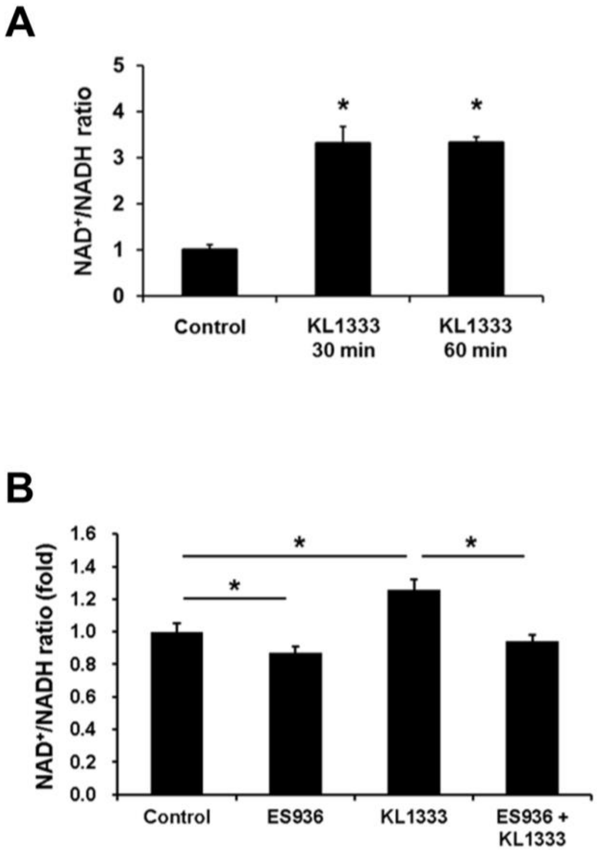

Figure 1. Effect of KL1333 on NAD$^+$/NADH ratio through NQO1. (A) HepG2 cells were treated with 2 μM KL1333 for the indicated times. **(B)** L6 myoblasts were pretreated with 250 nM ES936 for 1 hour, and then treated with 1 μM KL1333 for 30 minutes. Intracellular NAD$^+$ and NADH levels were measured, and NAD$^+$/NADH ratio was calculated based on the concentrations of NAD$^+$ and NADH. Error bars indicate ±SEM. *P<0.05.
